# A Callout for International Collaboration. Reply to Giger, E.V.; Tilen, R. Comment on “Shaniv et al. Neonatal Drug Formularies—A Global Scope. *Children* 2023, *10*, 848”

**DOI:** 10.3390/children10111803

**Published:** 2023-11-13

**Authors:** Dotan Shaniv, Anne Smits, Karel Allegaert, on behalf of the Neonatal Drug Formularies Group

**Affiliations:** 1 Pharmacy Services, Kaplan Medical Center (Clalit Health Services), Pasternak St., P.O. Box 1, Rehovot 76100, Israel; dotan.shaniv@mail.huji.ac.il; 2 Neonatal Intensive Care Unit, Kaplan Medical Center (Clalit Health Services), Pasternak St., P.O. Box 1, Rehovot 76100, Israel; 3 Department of Development and Regeneration, KU Leuven, 3000 Leuven, Belgium; anne.smits@uzleuven.be; 4 Neonatal Intensive Care Unit, University Hospitals Leuven, 3000 Leuven, Belgium; 5 Department of Pharmaceutical and Pharmacological Sciences, KU Leuven, 3000 Leuven, Belgium; 6 Department of Hospital Pharmacy, Erasmus MC, 3015 GD Rotterdam, The Netherlands

We are very grateful that the global-scope paper on neonatal drug formularies has received a relevant amount of interest from the readership of the journal [1]. This interest also resulted in some specific responses, either in the form of a formal letter as a reply from the Swiss neonatal formulary (SwissPedDose), or as an e-comment associated with the paper from the Ibero-American Society of Neonatology (SIBEN) about their Spanish-language formulary called NEOFARMA-SIBEN [1,2].

This strengthens our message that—since drug information specific to neonates is very commonly absent in drug labels—neonatal formularies are indeed essential for safe and effective pharmacotherapy in (pre)term neonates. While different legal and structured initiatives have been pursued to support pediatric drug labeling efforts as a strategy to improve pediatric pharmacotherapy, neonatal drug labeling has not come this far and for the most part is still lacking. Consequently, and despite these legal incentives and initiatives, neonates are still commonly treated with medicines that have neither been specifically registered, nor indicated, for this population [3]. These formularies should be known among and available to neonatal healthcare professionals.

The objective of this global-scope paper was therefore to identify neonatal formularies, explore (dis)similarities, and raise awareness of their existence and diversity. We intended to map and compare neonatal formularies on content, structure and workflow to inform the readership of the various formularies available and their differences in characteristics and content, to use them properly for the benefit of their patients [1]. In essence, we have carried this out based on a questionnaire completed by reference persons of these formularies, and a data extraction tool, intended to be applied to the top 10 most commonly administered drugs in neonates [1].

In their letter, Giger and Tilen rightfully mentioned that the SwissPedDose formulary initially missed the opportunity we provided to be included in the original paper. After becoming informed of the published work, they were very interested in bringing the information on their content, structure and workflow to the public domain [2]. We simulate this approach, as it contributes to the further mapping of existing neonatal formularies. The authors rightfully accepted the suggestion to use the framework as described in the supplemental information of the source document (questionnaire, Supplementary Material 1 of [1]), as this enables any reader to compare their formulary to the other neonatal formularies. The utilization of the extraction tool (Supplementary Material 2 of [1]) to visualize potential differences in the type and granularity of information for the top 10 most commonly administered drugs in neonates might have had an added benefit, and we encourage other initiatives to use both supplements for standardized reporting [1]. 

The e-comment letter of the NEOFARMA-SIBEN formulary is very well taken, as we indeed failed to identify their formulary based on the search strategy described in the initial paper. We acknowledge the shortcomings of the search strategy used, as already clearly mentioned in the original paper [1]. We encourage the SIBEN group to provide their information to facilitate a comparison to other formularies, preferably using the guidance provided in the Supplementary Materials of the original paper. 

We believe that the original paper and both responses provide further evidence on the diversity of neonatal formularies and their content, structure and workflow, and prove that this description of neonatal formularies is indeed valuable to all neonatal healthcare professionals all over the world. We can only hope that this global-scope initiative may also result in collaborations and interactions between the groups maintaining these formularies. 

In a setting of limited resources (‘money and people’) with emerging new workflows like the benefit–risk assessment of off-label drug use in children (BRAVO) framework or the NEODOSE approach as new approaches, collaboration is very likely needed to really improve the assessment of the available evidence and produce evidence-based data, subsequently converted into bedside clinically useful drug information for neonates [4,5]. Such workflows can be further supported via the integration of physiology-based pharmacokinetics to generate or support dosing recommendations [6].

In conclusion, the above-mentioned responses strengthen the global scope of neonatal formularies. We recommend that other remaining formularies also provide their information, preferably using the guidance provided in the Supplementary Materials of the original paper, and we hope that this will result in collaborations to further improve the quality of these formularies via the integration of newly emerging workflows and tools. 
